# Rufomycin Targets ClpC1 Proteolysis in Mycobacterium tuberculosis and M. abscessus

**DOI:** 10.1128/AAC.02204-18

**Published:** 2019-02-26

**Authors:** Mary P. Choules, Nina M. Wolf, Hyun Lee, Jeffrey R. Anderson, Edyta M. Grzelak, Yuehong Wang, Rui Ma, Wei Gao, James B. McAlpine, Ying-Yu Jin, Jinhua Cheng, Hanki Lee, Joo-Won Suh, Nguyen Minh Duc, Seungwha Paik, Jin Ho Choe, Eun-Kyeong Jo, Chulhun L. Chang, Jong Seok Lee, Birgit U. Jaki, Guido F. Pauli, Scott G. Franzblau, Sanghyun Cho

**Affiliations:** a Institute for Tuberculosis Research, College of Pharmacy, University of Illinois at Chicago, Chicago, Illinois, USA; b Department of Medicinal Chemistry & Pharmacognosy, College of Pharmacy, University of Illinois at Chicago, Chicago, Illinois, USA; c Center for Biomolecular Sciences, College of Pharmacy, University of Illinois at Chicago, Chicago, Illinois, USA; d Center for Nutraceutical and Pharmaceutical Materials, Myongji University, Cheoin-gu, Gyeonggi-do, Republic of Korea; e Division of Bioscience and Bioinformatics, College of Natural Science, Myongji University, Cheoin-gu, Gyeonggi-do, Republic of Korea; f Department of Microbiology, Chungnam National University School of Medicine, Daejeon, Republic of Korea; g Infection Control Convergence Research Center, Chungnam National University School of Medicine, Daejeon, Republic of Korea; h Department of Laboratory Medicine, Pusan National University Yangsan Hospital, Yangsan, Republic of Korea; i International Tuberculosis Research Center, Changwon, Republic of Korea

**Keywords:** ClpC1, *Mycobacterium abscessus*, *Mycobacterium tuberculosis*, cyclic peptide, rufomycin

## Abstract

ClpC1 is an emerging new target for the treatment of Mycobacterium tuberculosis infections, and several cyclic peptides (ecumicin, cyclomarin A, and lassomycin) are known to act on this target. This study identified another group of peptides, the rufomycins (RUFs), as bactericidal to M. tuberculosis through the inhibition of ClpC1 and subsequent modulation of protein degradation of intracellular proteins. Rufomycin I (RUFI) was found to be a potent and selective lead compound for both M. tuberculosis (MIC, 0.02 μM) and Mycobacterium abscessus (MIC, 0.4 μM). Spontaneously generated mutants resistant to RUFI involved seven unique single nucleotide polymorphism (SNP) mutations at three distinct codons within the *N*-terminal domain of *clpC1* (V13, H77, and F80). RUFI also significantly decreased the proteolytic capabilities of the ClpC1/P1/P2 complex to degrade casein, while having no significant effect on the ATPase activity of ClpC1. This represents a marked difference from ecumicin, which inhibits ClpC1 proteolysis but stimulates the ATPase activity, thereby providing evidence that although these peptides share ClpC1 as a macromolecular target, their downstream effects are distinct, likely due to differences in binding.

## Abstract (precis)

ClpC1 is an emerging new target for the treatment of Mycobacterium tuberculosis infections, and several cyclic peptides (ecumicin, cyclomarin A, and lassomycin) are known to act on this target. This study identified another group of peptides, the rufomycins (RUFs), as bactericidal to M. tuberculosis through the inhibition of ClpC1 and subsequent modulation of protein degradation of intracellular proteins.

## INTRODUCTION

In 2016, the World Health Organization (WHO) recognized tuberculosis (TB) as the number one single infectious killer worldwide (1), and this status was maintained through 2017 (2), confirming that TB remains a major public health threat. With the continued emergence of multidrug-resistant and extensively drug-resistant strains (MDR and XDR) of Mycobacterium tuberculosis, it is a priority to identify new cellular targets for the treatment of M. tuberculosis infections. An additional growing health threat is infections with nontuberculous mycobacteria (NTM) (3). NTM can cause pulmonary and disseminated infections that affect immunocompromised and immunocompetent patients equally (4). Rapidly growing mycobacteria, including Mycobacterium abscessus, are major pathogens for pulmonary infections caused by NTM infections (5). The most drug-resistant NTM infections are caused by M. abscessus bacteria (6), and there is an urgent need for new drug development to improve the treatment outcomes for NTM diseases (7).

**FIG 1:**
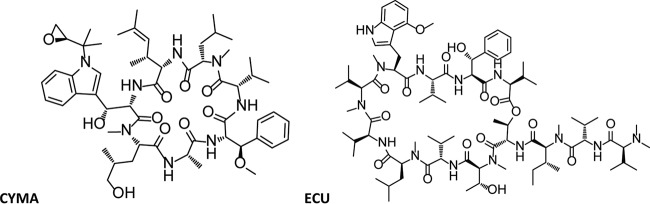
Structures of cyclomarin A (CYMA) and ecumicin (ECU).

Over the past decade, several compounds have been identified that inhibit ClpC1 (8), including cyclomarin A (CYMA) (9, 10), lassomycin (11), and ecumicin (ECU) (12). The structures of CYMA and ECU are shown in Fig. 1. ClpC1 is currently not targeted for the treatment of TB but has been established as a viable target for drug design (8, 12–14).

ClpC1 is the ATP-dependent homologue of the ClpC class of chaperone proteins present in M. tuberculosis (13) and is highly conserved among mycobacteria. Unlike in many other bacteria, ClpC proteins are essential for the viability of mycobacteria, especially M. tuberculosis (15). In M. tuberculosis, ClpC1 associates with the proteolytic domains, ClpP1 and ClpP2, and together they are responsible for waste protein degradation within the cell (16, 17). The proteolytic domains are strictly regulated by ClpC1 and capable of only low levels of unregulated protein degradation without the assistance of ClpC1. Therefore, without functional ClpC1, protein degradation within the cell is reduced or stopped completely. The downstream proteomic and metabolomic effects of ClpC1 perturbation are still being studied; however, two mechanisms have been proposed: (i) the uncoupling of ClpC1 activity from ClpP activity and (ii) the overactivation of ClpC1, leading to uncontrolled protein degradation (18). ECU has been found to trigger the first proposed mechanism by uncoupling ClpC1 from ClpP activity, leading to a decrease in protein degradation.

**FIG 2:**
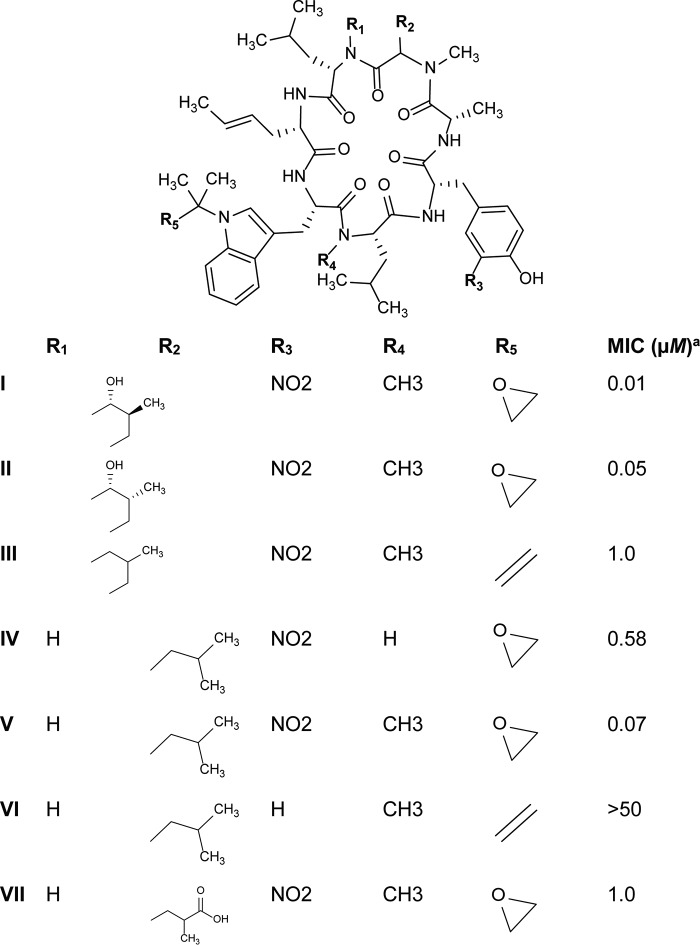
Rufomycin (RUF) analogues. *^a^*MIC against M. tuberculosis.

The goal of this project was to establish the rufomycins (RUFs) (19–22) (Fig. 2), which are also known as the ilamycins (23–25), as another class of compounds that target ClpC1. The RUFs were originally discovered in 1960 and patented by Takeda Chemical Industries. They were later patented with different claims by Eli Lilly and Company in 2000. The biosynthetic pathway was established by Tomita et al. in 2017 (24). The RUFs are a class of cyclic peptides containing seven amino acids and were found to be active against M. tuberculosis and M. abscessus. This report describes the mechanism of action of the RUF group through the inhibition of ClpC1 with the focus on rufomycin I (RUFI). In addition, this study demonstrated that RUFI is active in macrophages, acting against both M. tuberculosis and M. abscessus.

## RESULTS

### Isolation of rufomycin I.

RUFI was isolated from *Streptomyces* sp. strain MJM3502, determined to be 100% identical to Streptomyces atratus (NRRL B-16927) through classification using the 16S rRNA gene sequence (see the supplemental material). Strain MJM3502 was obtained by the Extract Collection of Useful Microorganisms (ECUM) at Myongji University, Republic of Korea, and was fermented in glucose-soybean starch (GSS) medium (rich medium). The culture medium supernatants were extracted with ethyl acetate and dried. MJM3502 was identified as a hit from the high-throughput screening (HTS) of approximately 7,000 actinomycete cultures as previously discussed (12). Briefly, RUFI was isolated by stepwise bioassay guided fractionation of the MJM3502 extract. Primary fractionation was performed using liquid-liquid separation with a biphasic mixture of dichloromethane (DCM), methanol (MeOH), and H_2_O. The lower layer was collected and dried prior to further separation using C_18_ flash chromatography with a gradient of acetonitrile (ACN) and H_2_O. The active fraction was subjected to preparative chromatography on a C_18_ silica gel using a 45% isocratic elution with ACN containing 0.1% formic acid (FA).

### Rufomycin has potent and selective *in vitro* activity against M. tuberculosis and M. abscessus.

**FIG 3:**
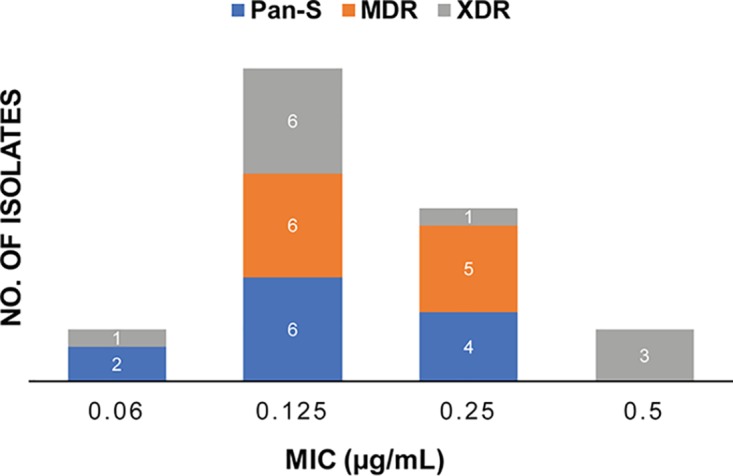
MIC distribution of RUFI against pan-susceptible (blue), MDR (orange), and XDR (gray) M. tuberculosis strains.

RUFI demonstrates potent and selective activity against M. tuberculosis, with MICs comparable or superior to those of standard first- or second-line drugs for the treatment of TB (Tables 1 and 2). RUFI also maintains activity against monoresistant, MDR, and XDR strains (Fig. 3), indicating that the RUFs most likely have a different molecular target than current drugs used in TB therapy. Additionally, RUFI retains its activity against strains from the five global M. tuberculosis clades representative of clinical TB disease across the world.

**TABLE 1:** MICs of RUFI and anti-TB drugs against M. tuberculosis

M. tuberculosis strain	MIC(s) (μM)^*a*^										
	RUFI	ECU	CYMA	RIF	INH	CAP	STR	PA824	BDQ	LZD	MXF
H_37_Rv	0.02	0.16	0.094	0.04	0.23	0.39	0.15	0.04	0.13	1.8	0.26
H_37_Rv resistant to drug											
RIF	0.038	0.19		>4	0.13	0.99	0.76	0.09			
INH	<0.004	<0.12		<0.016	>8	0.45	0.19	<0.031			
STR	0.078	<0.12		<0.016	0.10	3.6	>16	0.12			
MXF	0.047	0.31		0.027	0.24	0.67	0.50	<0.031			
KM	0.005	<0.12		<0.016	0.23	5.9	0.39	<0.031			
CS	<0.004	<0.12		<0.016	0.20	0.91	0.25	0.20			
CAP	0.061	0.29		0.054	0.124	>16	0.8	<0.031			
5 global clade representatives	0.003–0.011	0.13–0.38	0.012–0.079	0.01–0.027	0.25–0.47			0.03–0.10	0.017–0.11	0.41–1.6	0.06–0.12
rRUFI strains											
28 (H77R)	0.55	0.36	0.065	<0.016	0.22	0.47	0.13	<0.031	0.05	1.9	0.12
31 (F80S)	1.1	0.27	1.1	0.011	0.23	0.25	0.12	<0.031	0.015	0.94	0.082
35 (V13F)	1.7	0.18	0.17	<0.016	0.12	0.081	0.11	<0.031	<0.008	0.48	0.085
41 (F80V)	0.94	0.092	0.72	0.046	0.24	0.42	0.23	0.041	0.03	1.95	0.24
50 (F80I)	2.1	0.089	0.22	0.038	0.24	0.45	0.11	<0.031	0.06	1.72	0.24
51 (F80L)	0.22	0.34	0.23	0.025	0.23	0.46	0.14	0.046	0.06	1.75	0.23
53 (F80C)	1.2	0.19	1.1	0.031	0.24	0.39	0.12	<0.031	0.05	1.82	0.16
rECU strains											
1-5 (L92S)	<0.01	1.37	0.034	0.010	0.29	0.42	0.15	<0.031	0.086	2.5	0.18
1-6 (L96P)	<0.01	1.5	0.010	0.013	0.30	0.49	0.16	<0.031	0.041	1.9	0.15
1-9 (L92F)	0.017	0.40	0.027	0.025	0.59			<0.031	0.042	1.6	0.14

a RUFI, rufomycin I; ECU, ecumicin; CYMA, cyclomarin; RIF, rifampin; INH, isoniazid; CAP, capreomycin; STR, streptomycin; KM, kanamycin; CS, cycloserine; PA-824, pretomanid; BDQ, bedaquiline; LZD, linezolid; MXF, moxifloxacin.

**TABLE 2:** Spectrum of activity of RUFI

Target species	MIC (μM)^*a*^											
	RUFI	ECU	CYMA	AmphB	KETO	GEN	AMP	RIF	INH	PA824	CLO	BDQ
C. albicans	>10	>32	>10	2.6	0.98							
E. coli	>10	>32	>10			0.23	16					
S. aureus	>10	>32	>10			0.33	6.1					
M. smegmatis	0.073	1.7	1.6					>120	360		2.39	
M. abscessus	0.42	>63	>5					>4	>8	>8	9.23	0.96
M. chelonae	4.24	0.97	>5					>4	>8	>8	10	0.46
M. marinum	<0.02	0.95	0.58					0.050	>8	>8	0.34	0.29
M. bovis	<0.02	<0.2	<0.02					<0.02	0.48	0.10	<0.20	0.1
M. kansasii	0.04	4	1.11					0.15	3.84	4.7	<0.20	0.029
M. avium	1.75	0.35	4.85					0.16	>8	>8	<0.20	0.049

a AmphB, amphotericin B; KETO, ketoconazole; GEN, gentamicin; AMP, ampicillin; CLO, clofazimine.

**FIG 4:**
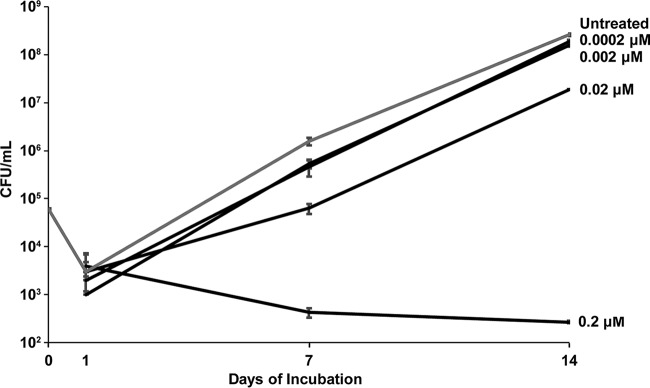
Rufomycin I (RUFI) has time- and concentration-dependent bactericidal activity against M. tuberculosis. The inoculum concentration was 6.0 × 10^4^ CFU/ml. Error bars represent the SDs of three measurements.

Much like for ECU, the inhibitory activities of RUFI and CYMA appear to be specific to mycobacteria, with no detected activity against Candida albicans, Escherichia coli, or Staphylococcus aureus (Table 2). Unlike ECU and CYMA, RUFI shows activity against all tested mycobacteria. Of special interest is its activity against M. abscessus, one of the more difficult-to-treat mycobacteria (26). RUFI has a clear concentration-dependent bactericidal activity against M. abscessus (MBC of 1.2 μM). The drastic difference in the potencies of the three compounds is most likely attributable to differences in physiochemical properties, but this remains unconfirmed. Moreover, RUFI has concentration- and time-dependent bactericidal activity against M. tuberculosis (minimum bactericidal concentration [MBC] of 0.4 μM) but appears to have a more bacteriostatic effect than that of ECU (Fig. 4). When a more concentrated bacterial inoculum was used, the degree of bacterial killing was reduced; therefore, the activity of RUFI could be bacterial inoculum concentration dependent, as is observed for isoniazid (INH). The difference in observed bacterial concentration from time zero (T_0_) to day 1 (T_1_) is most likely due to a lull in bacterial growth upon initial introduction to fresh bacterial media from frozen seed stock. Unfortunately, RUFI showed relatively low activity against nonreplicating cultures of M. tuberculosis (MIC, >10 μM; 75% inhibition at 10 μM).

**FIG 5:**
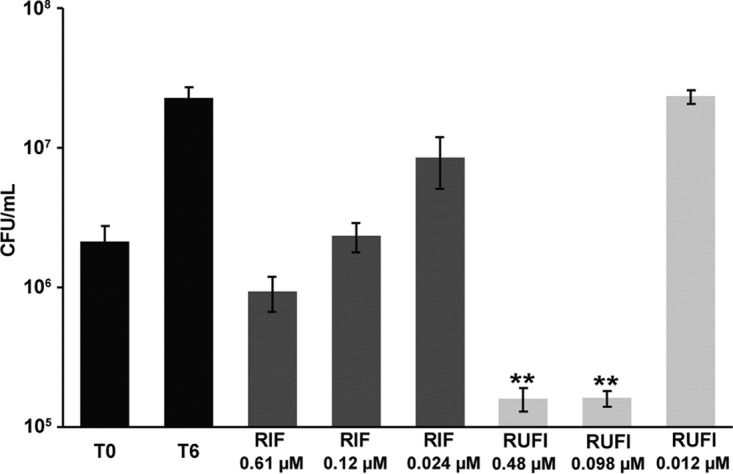
Activity of RUFI against M. tuberculosis in murine macrophages. Bars represent CFU prior to treatment (T_0_), no treatment (T_6_), and treatment with rifampin (RIF) or RUFI at the indicated concentrations. Values are means ± SDs from six measurements. According to the two-tailed *t* test, significant differences (*P* < 0.02 [**]) were observed between the untreated group (T_0_) and the groups treated with RUFI at 0.48 and 0.098 μM.

**FIG 6:**
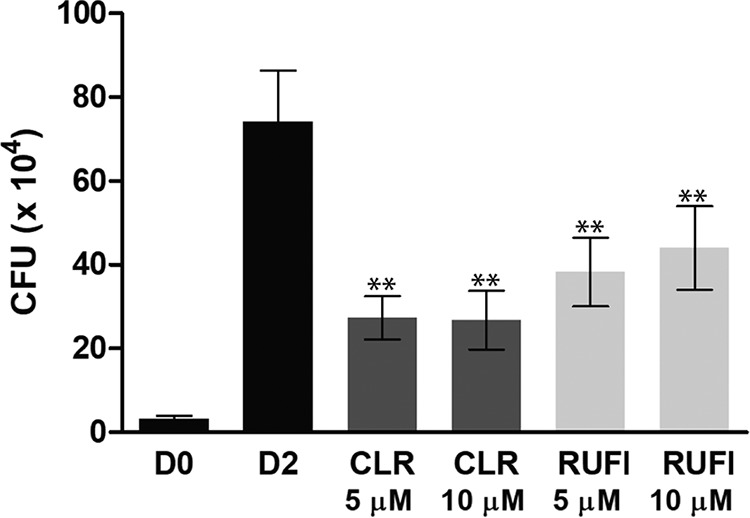
Activity of RUFI against M. abscessus in BMDMs. Bars represent CFU on the day of infection (D0), the second day with untreated cells (D2), and the second day for cells treated with clarithromycin (CLR) or RUFI at the indicated concentrations. Values are means ± SDs from six measurements with duplication. According to the two-tailed *t* test, significant differences (*P* < 0.001 [**]) were observed between D2 and all treatment groups.

As mycobacterial species are known to be capable of surviving in macrophages, RUFI was tested under these conditions. First, J774.1 cells were infected with M. tuberculosis, followed by RUFI treatment (Fig. 5). In this assay, RUFI was even more potent than rifampin (RIF), which is a clinically used first-line anti-TB drug. RUFI treatment also reduced the CFU of M. abscessus infected in bone marrow-derived macrophages (BMDMs) (Fig. 6). RUFI was as effective as clarithromycin (CLR), which is reported to effectively kill M. abscessus residing in macrophages. In short, significant antimicrobial effects of RUFI were observed in both M. tuberculosis and M. abscessus macrophage infections, indicating that RUFI is a potent antimycobacterial compound that can penetrate macrophages to eliminate intracellular mycobacteria.

RUFI was also found to act selectively on mycobacteria, as seen with the lack of toxicity to the Vero cell line. Although some toxicity was observed against the macrophage J774.1 cell line (Table 3), the concentration effecting a reduction in fluorescence of 50% relative to untreated cells (IC_50_) is very similar to that of bedaquiline (BDQ) and far above the MICs for mycobacteria. This gives RUFI a selectivity index (SI) of >2,500 (IC_50_/MIC) against M. tuberculosis and an SI of >100 against M. abscessus. Interestingly, unlike ECU, RUFI shows some cytotoxic activity against the melanoma cancer cell line MDA-MB-435, the breast cancer cell line MDA-MB-231, and the ovarian cancer cell line OVCAR 3. Studies addressing the mechanism of this cytotoxicity are ongoing and will be reported separately.

**TABLE 3:** Toxicity of RUFI and ECU against mammalian cell lines

Cell line	IC_50_ (μM)					IC_50_ (nM), paclitaxel^*a*^
	RUFI	CYMA	ECU	BDQ	RIF	
Vero	>50	>50	>63	27	120	
J774	13	19	>32	20	95	
MDA-MB-435^*b*^	12		>25			0.1
MDA-MB-231^*c*^	7.7		>25			170
OVCAR 3^*d*^	14		>25			1.5

a Originally named taxol.

b Human melanoma cell line.

c Human breast cancer cell line.

d Human ovarian cancer cell line.

### Rufomycin-resistant M. tuberculosis strains harbor mutations in *clpC1*.

Spontaneously generated mutants resistant to RUFI (rRUFI) were selected for *in vitro* testing through stepwise inoculation on 7H11 agar plates impregnated with increasing concentrations of RUFI as described in Materials and Methods. Single-step mutations were selected at a frequency of 2.6 × 10^−9^. A total of 34 colonies were selected and determined by PCR and Sanger DNA sequencing to contain a single nucleotide polymorphism (SNP) within *clpC1* (Table 4). The strains were tested against a panel of standard antibiotics to determine phenotypic specificity to RUFI (Table 1). Based on the level of selective resistance to RUFI, defined as a greater than 4× increase in MIC compared to that for H_37_Rv, seven strains (strains 28, 31, 35, 41, 50, 51, and 53) containing unique SNP mutations within *clpC1* were selected for confirmatory whole-genome sequencing (WGS). All strains were confirmed to harbor a nonsynonymous mutation in *clpC1* (Rv3596c) compared to M. tuberculosis H_37_Rv. Each strain contained one of seven mutations in the N-terminal domain of ClpC1 (10, 12, 13). Similar to what has been reported for ECU (12), multiple mutation sites were identified within the genome of all rRUFI strains relative to H_37_Rv. Most minor mutations were attributed to genetic drift and therefore determined not to be associated with the molecular target. A few mutations were present in only one of the seven rRUFI strains (53, *fadE22* [Rv3061c]; 51, Rv3438, *mce4F* [Rv349c], and *fdxB* [Rv3554]; and 35, *papA2* [Rv3820c]); however, these mutations were in nonessential genes, and their relation to the function of ClpC1 is unclear at this time. The most common mutations occurred at residue 80, resulting in a replacement of phenylalanine (F) by isoleucine (I), valine (V), serine (S), cysteine (C), or leucine (L). Substitutions of arginine (R) for histidine (H) at residue 77 and phenylalanine for valine at residue 13 were also observed.

**TABLE 4:** Mutations of clpC1 in RUFI-resistant strains

Resistant strain	Codon mutation	Residue substitution
3	TTT → ATT	F80I
4	TTT → ATT	F80I
5	TTT → ATT	F80I
6	TTT → GTT	F80V
7	TTT → CTT	F80L
8	TTT → ATT	F80I
11	CAC → CGC	H77R
12	TTT → GTT	F80V
13	TTT → GTT	F80V
15	TTT → ATT	F80I
18	TTT → ATT	F80I
20	CAC → CGC	H77R
21	TTT → ATT	F80I
23	TTT → CTT	F80L
25	TTT → ATT	F80I
28	CAC → CGC	H77R
29	TTT → GTT	F80V
31	TTT → TCT	F80S
34	TTT → ATT	F80I
35	GTC → TTC	V13F
37	TTT → GTT	F80V
38	TTT → ATT	F80I
39	TTT → GTT	F80V
40	TTT → CTT	F80L
41	TTT → GTT	F80V
43	TTT → ATT	F80I
45	TTT → CTT	F80L
46	TTT → GTT	F80V
48	TTT → ATT	F80I
49	TTT → ATT	F80I
50	TTT → ATT	F80I
51	TTT → CTT	F80L
53	TTT → TGT	F80C
54	TTT → GTT	F80V

When compared to the spontaneously generated mutations in *clpC1* conferring resistance to ECU (L92S, L92F, and L96P) (12) and genetically generated mutants with resistance to CYMA (F2A, F2Y, F80A F80Y, E89A, and E89Q) (10), only alterations of residue 80 were found to be in common. To further test the significance of the observed mutations, ECU, CYMA, and RUFI were tested against M. tuberculosis mutants resisant to RUFI and ECU (Table 1). RUFI and CYMA both maintained their activity against ECU-resistant strains, while ECU also maintained its activity against mutants resistant to RUFI, indicating a lack of cross-resistance. On the other hand, mutants resistant to RUFI harboring the SNP mutation at residue 80 showed resistance to CYMA, while retaining activity against mutants with SNP mutations at residues 77 and 13. The increase from baseline in the MIC of both RUFI and CYMA against M. tuberculosis strains containing a mutation at residue 80, while ECU maintained its full activity, suggests that RUFI might bind to ClpC1 in a fashion similar to that of CYMA but different from that of ECU. This hypothesis is supported by preliminary data indicating antagonistic effects when dosing these compounds in combination, which could be the result of competitive binding effects between the ClpC1 inhibitors.

### Rufomycin inhibits proteolytic activity of the ClpP1P2 complex.

**FIG 7:**
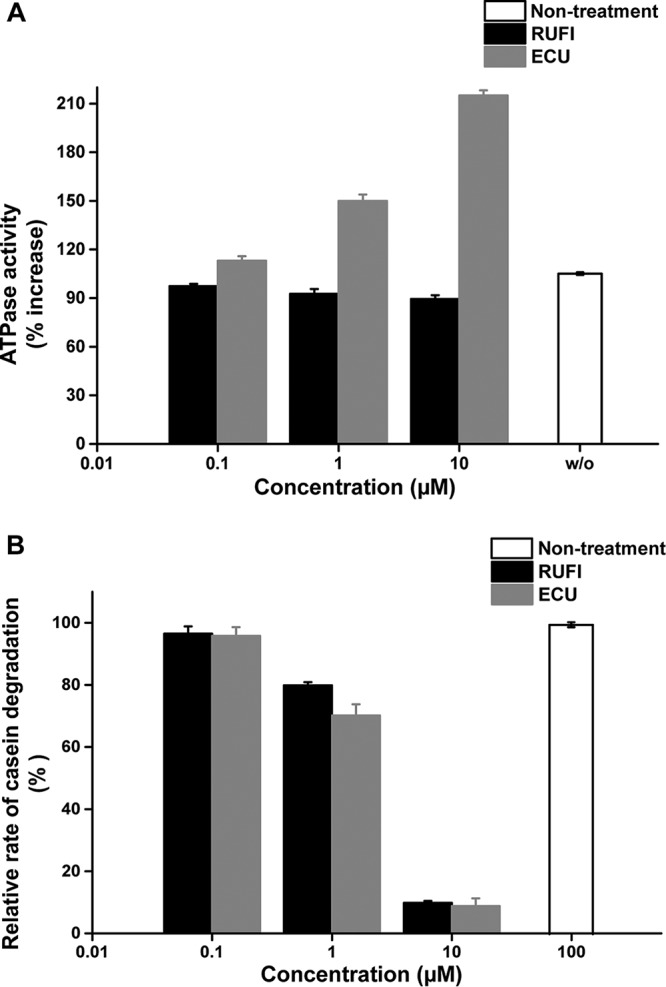
ClpC1 ATPase activity (A) and proteolytic activity of the ClpC1/P1/P2 complex (B) in response to ECU and RUFI treatment. This experiment was carried out in triplicate.

To clarify the mode of action of RUFI against M. tuberculosis, the compound was subjected to several functional assays previously established to assess the inhibitory capacity of compounds against ClpC1 and the associated proteolytic complex, ClpC1/P1/P2 (Fig. 7) (27–29). The first assay (Fig. 7A) assessed the effect of RUFI on the ATP-dependent ATPase activity of ClpC1. Unlike ECU, which stimulates ATPase activity, resulting in the uncoupling of ATPase activity from proteolytic activity, RUFI had a mild and insignificant inhibitory effect on ClpC1 ATPase activity. This further solidifies the hypothesis that RUFI and ECU affect ClpC1 differently.

The second assay (Fig. 7B) aimed to assess the effect RUFI has on the ability of ClpC1 to stimulate ATP-dependent degradation of the model substrate, fluorescein isothiocyanate (FITC)-casein, by the complex ClpC1/P1/P2. Comparable to ECU, RUFI strongly inhibited the proteolytic capabilities of the ClpC1/P1/P2 complex to degrade casein. This was found to be statistically significant (*P* < 0.01) at a concentration of 10 μM. The ability of RUFI to inhibit the ATP-dependent selection and subsequent degradation of proteins within the cell can explain the observed cytotoxic effect of RUFI on mycobacteria. This observed inhibitory effect further confirms the cellular target of RUFI and the selectivity of RUFI for mycobacteria.

### RUFI binds to mycobacterial ClpC1.

**FIG 8:**
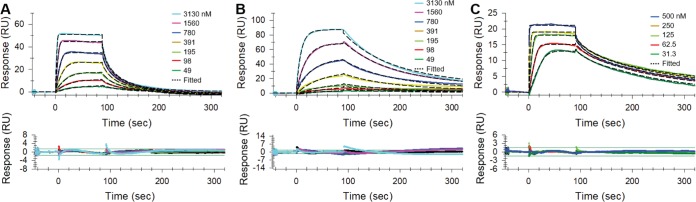
Binding of RUFI (A), ECU (B), and CYMA (C) to wild-type full-length ClpC1 from M. abscessus.

RUFI, ECU, and CYMA were tested against M. tuberculosis, M. abscessus, and Mycobacterium chelonae in surface plasmon resonance (SPR) binding assays (Fig. 8; Table 5). ClpC1, especially the *N*-terminal domain (NTD), is highly conserved among mycobacteria (30). The ClpC1 constructs tested were found to be 100% genetically identical in the NTD but varied by several residues in other regions of the protein. The primary sequences of M. abscessus and M. chelonae have a 99.1% identity, while each has a 91.8% identity to the primary sequence of M. tuberculosis. The M. abscessus ClpC1 used for experiments was found to contain one residue different from the bacterial strain used for *in vitro* assays; however, this difference was not present in the NTD. The strain used in SPR contains a G→S mutation near the C terminus at residue 811.

**TABLE 5:** Affinities of binding of cyclopeptides to full-length ClpC1 determined by SPR^*a*^

Target strain	Inhibitor	*k*_a_ (1/Ms)	*k*_d_ (1/s)	*K_D_* (nM)
M. tuberculosis	RUFI	2.98 × 10^6^ ± 1.8%	3.01 × 10^−1^ ± 1.7%	101 ± 1.8
	ECU	6.12 × 10^4^ ± 0.7%	6.14 × 10^−3^ ± 5.4%	100 ± 4.8
	CYMA	4.36 × 10^6^ ± 1.8%	2.48 × 10^−2^ ± 1.5%	5.69 ± 0.09
M. abscessus	RUFI	1.42 × 10^6^ ± 1.3%	1.37 × 10^−1^ ± 5.6%	96.4 ± 3.3
	ECU	5.66 × 10^4^ ± 4.7%	7.01 × 10^−3^ ± 0.4%	125 ± 3.2
	CYMA	5.68 × 10^6^ ± 1.5%	2.19 × 10^−2^ ± 1.3%	3.85 ± 0.05
M. chelonae	RUFI	2.96 × 10^5^ ± 0.6%	2.13 × 10^−2^ ± 0.4%	72.1 ± 0.4
	ECU	5.34 × 10^4^ ± 2.9%	6.72 × 10^−3^ ± 1.3%	126 ± 2.7
	CYMA	7.60 × 10^6^ ± 14%	2.45 × 10^−2^ ± 13%	3.23 ± 0.44

a Percent standard deviations were calculated from two or three independent experiments.

Although overall binding affinities of RUFI and ECU to ClpC1 in equilibrium are similar, association and dissociation rates are very different. Both rates of RUFI are higher, at 1.42 × 10^6^ M^−1^s^−1^ and 1.37 × 10^−1^ s^−1^, respectively, than those of ECU, at 5.66 × 10^4^ M^−1^s^−1^ and 7.01 × 10^−3^ s^−1^, respectively (Fig. 8; Table 5). This indicates that RUFI recognizes and binds to ClpC1 much faster and dissociates faster as well, whereas ECU binds slowly and remains bound much longer. The association rate of CYMA seems to be ∼4-fold higher (5.68 × 10^6^ M^−1^s^−1^) than that of RUFI, whereas the dissociation rate of CYMA is ∼7-fold lower (2.19 × 10^−2^ s^−1^) than that of RUFI and ∼2.7-fold higher than that of ECU to the full-length ClpC1 from M. abscessus. In the case of M. tuberculosis full-length ClpC1, association rates of RUFI and CYMA were very similar, so the overall binding affinity differences mainly came from the ∼13-fold-lower dissociation rate of CYMA than that of RUFI. Collectively, this indicated that the equilibrium binding affinity of CYMA is tighter than those of both RUFI and ECU. The binding affinities for all three compounds were found to be similar for the tested mycobacterial ClpC1 proteins. This is consistent with the observation that these compounds bind the NTD of ClpC1. The lack of significant difference in binding affinity also indicates that the differences in full-length (FL) sequence have only a minimal effect on the binding of ECU, RUFI, or CYMA. However, this does not explain the difference in activity observed *in vitro*. Therefore, the difference in activity is most likely explained by other cellular factors, such as cellular permeation, enzymatic deactivation, or efflux pumps.

### Rufomycin I PK study.

**FIG 9:**
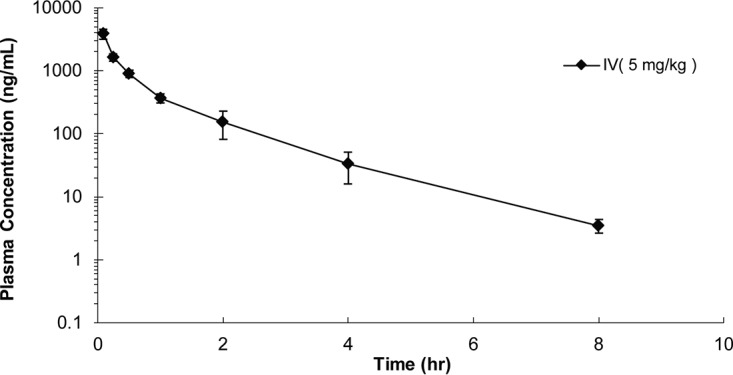
Mean plasma concentrations of RUFI in mice (*n* = 3) after a single i.v. dose of RUFI at 5 mg/kg.

Following a single intravenous (i.v.) administration of 5 mg/kg of body weight, RUFI showed behavior *in vivo* similar to that of ECU (12). Although the formulation {10% EtOH, 40% 10% (2-hydroxypropyl)-β-cyclodextrin [HP-β-CD] solution, 50% polyethylene glycol 400 [PEG 400]} was different from the micelle formulation used for ECU, RUFI was eliminated from the plasma with a mean (*n* = 3) terminal half-life of 1 h (Fig. 9). This was less than the 3 h observed for ECU. The maximum plasma concentration (*C*_0_) was determined to reach 5.1 to 7.4 μg/g, with area under the curve from 0 h to infinity (AUC_0–∞_) determined to be 1.8 to 2.4 μg/g. This observed difference from ECU can most likely be explained by the difference in formulation, as micellar formulations tend to improve pharmacokinetic (PK) properties of lipophilic compounds. Additionally, there were no clinical abnormalities observed in any of the mice tested.

## DISCUSSION

This report describes another potent, narrow-spectrum anti-TB cyclic peptide isolated from an actinomycete as a result of an HTS program against M. tuberculosis. This compound, RUF1, also happens to bind to the same molecular target, ClpC1, as ECU but with a different mechanism of action, thereby offering opportunities to further explore the complexities of ClpC1 as a new drug target. As chaperone protease complexes are present in many bacterial families, and an ideal antimycobacterial agent has a narrow spectrum of activity due to the long treatment duration, this study assessed the selectivity of RUFI against several nonmycobacterial species. RUFI was determined to be selective to mycobacteria. With the expectation of targeting MDR and XDR M. tuberculosis, the ability for RUFI to maintain activity against these strains is essential. RUFI maintained activity against all tested monoresistant, MDR, and XDR strains of M. tuberculosis, suggesting that RUFI would be an ideal drug lead for MDR and XDR TB treatment. Based on the reported cytotoxicity of the structurally related anti-TB ohmyungsamycins A and B (31, 32) to several cancer lines, RUFI and ECU were also tested against several cancer cell lines to assess their potential as chemotherapy agents. While ECU lacked significant cytotoxic activity, RUFI demonstrated some toxicity against the tested cancer cell lines while demonstrating no toxicity to Vero cells, indicating an area for further exploration.

Although both ECU and RUFI target ClpC1, their binding and resulting downstream effects are likely to be different, as indicated by WGS of resistant mutants, SPR, and functional assays. This further increases the interest in ClpC1 as a future target for TB drug treatment. The lack of cross-resistance between rECU and rRUFI strains indicates the potential for a broader range of treatment options for resistant and MDR-TB patients. For example, if a patient begins to show resistance to one ClpC1 inhibitor, this resistance may not necessarily preclude the use of a different compound from the same class. The ability of RUFI and ECU to significantly inhibit cellular protein degradation by the ClpC1/P1/P2 complex, as observed in the functional assays, poses additional questions about the downstream effects of these compounds. Although RUFI appears to be more potent (MIC of 0.02 μM) than ECU (MIC of 0.16 μM), the bactericidal effect of ECU (MBC of 0.34 μM) is roughly equal to that of RUFI (MBC of 0.40 μM). In addition, ECU shows activity against nonreplicating cultures (MBC of 1.5 μM), while RUFI apparently lacks this activity. Remarkably, the ECU analogues showed only minimal differences in MIC (33), while the MIC values of the RUF analogues varied greatly with small structural changes, as summarized in Fig. 2. The differences in the biological profiles of these two groups of cyclic peptides suggest that although they bind to the same molecular target, the specific binding patterns and the downstream effects on M. tuberculosis may differ greatly. Studies to observe their effects on ClpC1 and the proteomic consequences of these inhibitors are ongoing.

Finally, and important for further preclinical drug development, RUFI offers a lower-molecular-weight alternative to ECU with potentially less solubility and cellular penetration issues. This is supported by the observed activity of RUFI against M. abscessus versus the lack of activity of ECU, which could not be explained by SPR binding affinity assays. Additionally, the PK behavior of RUFI suggests that this compound may behave similarly to ECU in efficacy-based studies, as both compounds have reasonable serum half-lives and clearance (12). This also leads to the additional application of RUFI as a drug candidate for the treatment of M. abscessus, which often causes skin and soft tissue as well as pulmonary infections (34, 35). Among rapidly growing NTM strains, M. abscessus is considered one of the most virulent and resistant to antimicrobial therapy, thus having limited therapeutic options (26). The present data show the unique potential of RUFI as a drug lead against NTM infections, including M. abscessus infections.

Even though the challenges associated with oral dosing appear to be similar for all investigated cyclic peptides, determining the structural pharmacophore common to RUFI and ECU with the assistance of SAR studies could lead to an improved drug candidate. In addition, efforts to improve the bioavailability of cyclic peptides are gaining traction as their pharmaceutical applications become more apparent (36–41). In summary, this study coupled with others (8–12, 42) demonstrates that ClpC1 is a viable drug target for the treatment of TB.

## MATERIALS AND METHODS

### MICs versus M. tuberculosis.

All experiments with M. tuberculosis were conducted within a biosafety level 3 (BSL3) laboratory. The microplate alamarBlue assay (MABA) was used to determine MIC values as previously described (43). Briefly, samples were dissolved in dimethyl sulfoxide (DMSO) prior to addition to the assay plate containing Middlebrook 7H12 medium (Sigma-Aldrich, Darmstadt, Germany). Twofold serial dilution of the compounds was performed. Standard control compounds used were rifampin (RIF), isoniazid (INH), linezolid (LZD), moxifloxacin (MXF), pretomanid (PA-824), bedaquiline (BDQ), capreomycin (CAP), clofazimine (CLO), streptomycin (STR), kanamycin (KM), and cycloserine (CS). All standard compounds were obtained from Sigma-Aldrich except BDQ and PA-824, which were obtained from The Global Alliance for TB Drug Development. Plates were inoculated with M. tuberculosis strain H_37_Rv (ATCC 27294) and incubated for 7 days at 37°C. Then the redox dye (20 μl of 0.6 mM resazurin dye and 12 μl of 20% Tween 80) was added to each well, and the plates were incubated for an additional 18 to 24 h at 37°C. Final fluorescence was measured at 530 nm excitation and 590 nm emission using either a CLARIOstar (BMG LABTECH, Ortenberg, Germany) or VICTOR X3 (PerkinElmer Inc., Waltham, MA) plate reader. The MIC was defined as the minimum concentration of the compound required to achieve a reduction in fluorescence of 90% relative to untreated bacterial controls.

### Cytotoxicity in mammalian cells.

Cytotoxicity was tested using the Vero cell (ATCC CRL-81) and J774A.1 macrophage (ATCC TIB-67) lines (44–46). Vero cells were cultured in Eagle’s minimum essential medium (MEM) containing 10% fetal bovine serum (FBS) plus penicillin and streptomycin. Vero cells were prepared and washed with 0.25% trypsin-EDTA 1× solution in Hanks’ balanced salt solution (HBSS; pH 7.4). J774 cells were cultured in Dulbecco’s modified Eagle’s medium (DMEM) containing 10% FBS plus polymyxin B. J774 cells were detached by cell scraper. After verifying the morphology by microscopy and adjusting the density to 3 to 5 × 10^5^ cells/ml in MEM, 100 μl of the cell suspension was incubated with the test compounds at 37°C for 72 h; visual inspection was performed after 24 h. Then 20 μl of 0.6 mM resazurin was added into each well and incubated for 4 h. The fluorescence was measured at excitation/emission wavelengths of 530/590 nm. The concentration of test compound effecting a reduction in fluorescence of 50% relative to untreated cells (IC_50_) was calculated. Cytotoxicity assessment was repeated using the J774A.1 macrophage line for the interpretation of antimycobacterial activity within the macrophage. RIF and BDQ were included as controls.

### Cytotoxicity against selected cancer cell lines.

Human melanoma cancer cells (MDA-MB-435), human breast cancer cells (MDA-MB-231), and human ovarian cancer cells (OVCAR3) were purchased from the American Type Culture Collection (Manassas, VA). Each cell line was propagated at 37°C in 5% CO_2_ in RPMI 1640 medium supplemented with FBS (10%), penicillin (100 U/ml), and streptomycin (100 μg/ml). Cells in log phase were harvested by trypsinization, followed by two washing cycles to remove all traces of enzyme. A total of 5,000 cells were seeded per well of a 96-well clear, flat-bottom plate (Microtest 96; Falcon) and incubated overnight (37°C in 5% CO_2_). Samples dissolved in DMSO were then diluted and added to the respective wells. The cells were incubated in the presence of test substance for 72 h at 37°C and evaluated for viability with a commercial absorbance assay (CellTiter 96 AQ_ueous_ One Solution cell proliferation assay; Promega Corp., Madison, WI) that measured viable cells. IC_50_ values are expressed in micromolar values relative to the solvent (DMSO) control.

### MICs against mono-drug-resistant isolates.

MICs were assessed against strains of M. tuberculosis H_37_Rv with mono-drug resistance to RIF (ATCC 35838), INH (ATCC 35822), STR (ATCC 35820), KM (ATCC 35827), CS (ATCC 35826), MXF, and CAP. The rMXF and rCAP strains were isolated in our laboratory and contained mutations within *gyrA* and *tylA*, respectively. The assays were carried out with the same protocol as used for susceptible H_37_Rv strains.

### MICs against susceptible, MDR, and XDR clinical strains.

Twelve pan-susceptible, eleven MDR, and eleven XDR (total, 34 strains) clinical isolates strains were used. All strains were isolated from patients who were enrolled in a prospective observational cohort study (ClinicalTrials.gov identification number NCT00341601) between 2005 and 2012, and the strains were stored in −70°C deep freezer at the International Tuberculosis Research Center (ITRC; Masan, South Korea) before use. All experiments utilizing M. tuberculosis were performed in a BSL3 laboratory in ITRC. Phenotypic drug susceptibility testing was performed by using the Bactec MGIT 960 system according to the manufacturer’s recommendations (47) with the following final drug concentrations: 0.1 μg/ml for INH, 1.0 μg/ml for RIF, 5.0 μg/ml for ethambutol (EMB), 1.0 μg/ml for STR, 2.5 μg/ml for KM, 2.5 μg/ml for CAP, 2.0 μg/ml for ofloxacin (OFX), and 0.25 μg/ml for MXF. For the detection of genotypes, all isolates were subjected to whole-genome sequencing; a paired-end sequencing library was constructed with a 500-bp insert size using the NexTera sample preparation kit (Illumina, San Diego, CA) for the Illumina-HiSeq platform, and genotypic drug resistance characteristics for INH and RIF were identified using PhyResSE version 1.0 (bioinf.fz-borstel, Germany).

When performing the susceptibility testing with RUFI, INH and RIF were included to confirm the validity of the test results. The drug susceptibility testing was performed by using the Bactec MGIT 960 system according to the manufacturer’s recommendations (47) with the following final drug concentrations: 0.1 μg/ml for INH, 1.0 μg/ml for RIF, and 0.03, 0.06, 0.125, 0.25, 0.5, and 1.0 μg/ml for RUFI.

### MICs against nonmycobacteria.

MICs, defined as the lowest concentration resulting in ≥90% inhibition of bacterial growth relative to bacterial control against the nonmycobacterial strains Escherichia coli (ATCC 25922) and Staphylococcus aureus (ATCC 29213), were determined by measuring optical density at a wavelength of 570 nm (OD_570_) after 16 h of incubation at 37°C in 2.2% Mueller-Hinton II broth (CAMH; Becton, Dickinson, Sparks, MD). Standard compounds used were ampicillin (AMP) and gentamicin (GEN). The MIC against Candida albicans (ATCC 10231) was determined after 48 h of incubation at 37°C by measuring OD_570_ in 1% Cellgro RPMI 1640 medium (Mediatech Inc., Manassas, VA) supplemented with 1.8% d-(+)-dextrose (ICN Biomedicals, Aurora, OH) and 3.5% morpholinopropanesulfonic acid (MOPS) (Acros, NJ). Amphotericin B (AmphB) and ketoconazole (KETO) were used as standard compounds.

### MICs against nontuberculous mycobacteria.

MICs against M. abscessus (ATCC 19977) and M. smegmatis (ATCC 700084) were determined after 72 h of incubation at 37°C followed by addition of 0.6 mM resazurin and 20% Tween 80 and an additional 4 h of incubation. M. chelonae (ATCC 35752) was incubated for 72 h, and M. marinum (ATCC 927) for 120 h, both at 30°C. The MIC against M. bovis (ATCC 35734) was determined with the MABA protocol. M. kansasii (ATCC 12478) and M. avium (ATCC 15769) were incubated at 37°C for 6 and 7 days, respectively. All strains were cultured using Middlebrook 7H9 medium supplemented with 10% oleic acid-albumin-dextrose-catalase (OADC).

### MICs against genetically diverse M. tuberculosis.

MIC values were determined by the MABA protocol against a panel of M. tuberculosis isolates that were collected from around the world and grouped into six major single nucleotide polymorphism (SNP) clusters, five of which were tested (48). Tested isolates were X001354 from the East African Indian lineage, X004439 and X004244 from the East Asian lineage, and X005282 and X005319 from the Euro-American lineage. X001354, representing the Indo-Oceanic lineage, was not tested.

### MBC against M. tuberculosis and M. abscessus.

The minimum bactericidal concentration (MBC) against M. tuberculosis was determined for RUFI and the standard compound RIF following the MABA protocol, with each compound run in triplicate. Two sets of plates were run in parallel; after the 7-day incubation period, one plate was analyzed following MABA protocol, with the addition of resazurin and Tween 80, while no dye was added to the second plate. Instead, the contents of the inoculated test wells were collected into 1.5-ml tubes, washed with phosphate-buffered saline (PBS), and then plated on 7H11 agar. Plates were incubated at 37°C for 2 to 3 weeks before CFU determination. The MBC was defined as the lowest concentration reducing CFU by 99% relative to the time-zero inoculum.

The MBC against M. abscessus was determined for RUFI and the standard compound BDQ by following the MABA protocol, with a 72-h incubation in 7H9 medium supplemented with OADC. Parallel plates were processed as described for M. tuberculosis. The plates were incubated at 37°C for 5 days before CFU determination. The MBC was defined as the lowest concentration reducing CFU by 99% relative to the time-zero inoculum.

### Activity in macrophage culture.

Inhibition of M. tuberculosis Erdman (ATCC 35801) in macrophage culture was assessed as previously described (12, 45, 49). Briefly, J774A.1 cells were grown, adjusted to a concentration of 1 × 10^5^ to 3 × 10^5^/ml, and then distributed to 13-mm coverslips in 24-well plates to facilitate the washing out of nonphagocytosed M. tuberculosis. Cells were then incubated at 37°C with 5% CO_2_ overnight prior to infection with M. tuberculosis. The M. tuberculosis inoculum was prepared at a concentration of 1 × 10^5^ to 3 × 10^5^ CFU/ml in DMEM and transferred to a fresh 24-well plate. J774A.1 coverslips were transferred to the 24-well plate containing the M. tuberculosis, and the plate was incubated for 1 h at 37°C with 5% CO_2_. Next bacteria were washed from the cell coverslips with HBSS, and then fresh DMEM was added to the wells. After the plates were incubated overnight to allow phagocytosis, they were washed three times with warm HBSS and transferred to a fresh 24-well plate containing 1 ml of diluted compound. RUFI was tested at 0.019, 0.098, and 0.48 μM versus the control compound, RIF, at 0.024, 0.12, and 0.61 μM. The plates were then incubated for 6 days at 37°C and 5% CO_2_. All experiments were run in triplicate. For the time-zero (T_0_) untreated control and the other cultures after 6 days of incubation, macrophages were removed, subjected to lysis with 200 μl of 0.25% sodium dodecyl sulfate (SDS), and incubated at 37°C for 10 min. Fresh medium was then added, and samples were sonicated, diluted, plated on 7H11 agar, and incubated at 37°C. The CFU were determined following 2 to 3 weeks of incubation.

### Intracellular survival assay in BMDMs.

Bone marrow-derived macrophages (BMDMs) were isolated from the femurs and tibias of C57BL/6 mice (5 to 6 weeks old) and differentiated by culturing for 4 days in DMEM containing 10% (vol/vol) FBS and 25 ng/ml of macrophage colony-stimulating factor (M-CSF; R&D, Minneapolis, MN). The final densities of differentiated BMDMs were 1 × 10^5^ to 3 × 10^5^ per well in 48-well plates. Cells were infected for 2 h with M. abscessus at a multiplicity of infection (MOI) of 5. Next cells were washed with PBS and incubated in DMEM containing 50 μg/ml of gentamicin (Sigma-Aldrich). RUFI and clarithromycin (CLR) were added to the media to achieve final concentrations of 5 and 10 μM, respectively, and then the cultures were incubated for 2 days. The supernatant was removed, and cells were lysed in sterile distilled water for 30 min at 37°C. Cell lysates were serially diluted, plated on 7H10 agar, and incubated at 37°C for 2 to 3 days for enumeration of CFU.

### Selection of rufomycin-resistant strains of M. tuberculosis.

M. tuberculosis mutants resistant to RUFI were generated by plating H_37_Rv on 7H11 agar plates containing 1 μg/ml and 5 μg/ml of RUFI. Plates were prepared by adding the appropriate volume of RUFI in DMSO to molten 7H11 medium. A total of 4 × 10^9^ CFU/ml of M. tuberculosis were plated on the agar plates and incubated for 3 to 4 weeks at 37°C. Colonies were then streaked on 7H11 agar plates containing 5 μg/ml and 10 μg/ml of RUFI and incubated for 3 to 4 weeks at 37°C. This process was repeated for 10 μg/ml and 20 μg/ml and then 20 μg/ml and 40 μg/ml. Colonies that still grew at the highest concentration of RUFI were picked for inoculation in liquid 7H9 medium containing 0.001× MIC of RUFI and incubated at 37°C for 1 to 2 weeks. The harvested cells were filtered using an 8.0-μm sterile filter, washed with PBS, and stored at −80°C. The MIC determination against these strains was done using the MABA protocol except that plates were incubated for 10 days prior to the addition of resazurin and Tween 80.

### Whole-genome sequencing of rufomycin-resistant M. tuberculosis.

Genomic DNAs of the rRUFI M. tuberculosis strains (#28, 31, 35, 41, 50, 51, and 53) were isolated as described previously (50). Genomic DNA extracted from each of these M. tuberculosis strains and from wild-type H_37_Rv was sequenced on a HiSeq 2000 instrument (Illumina, San Diego, CA). The raw sequence data were imported into the CLC Genomics Workbench software package (Qiagen, Redwood City, CA; v5.5) as paired reads. Stringent trimming was performed (0.01 quality trimming threshold with no degeneracies allowed), and all sequences shorter than 100 bases after trimmings were discarded. After trimming, approximately 5 to 11.5 million reads were recovered from each sample. These reads were mapped to the reference genome of M. tuberculosis H_37_Rv (GenBank accession no. NC_000962) using default assembly parameters (0.5 length fraction and 0.8 similarity fraction) implemented within CLC Genomics Workbench. The variants relative to the reference genome, including single nucleotide variants as well as insertions and deletions, were detected using the probabilistic variant detection routine within CLC Genomics Workbench. The parameters included a minimum coverage of 50× and a minimum variant probability of 50%. The reads from each strain were inspected with IGV software (Broad Institute) to identify any mutations missed due to coverage gaps and low read scores. PCR, Sanger DNA sequencing, and Sequencher (Gene Codes, Ann Arbor, MI) were used to confirm nonsynonymous variants.

### Mycobacterium *clpC1* expression and purification for surface plasmon resonance (SPR).

The coding sequence of *clpC1* (Rv3596c) was amplified by PCR from genomic DNA of the wild-type M. tuberculosis strain H_37_Rv and ligated to create a recombinant pET-30a(+) plasmid (Novagen EMD Millipore, Darmstadt, Germany) with C-terminal His tag. The wild-type *clpC1* PCR fragment was cloned into the NdeI and HindIII restriction enzyme (RE) sites of pET-30a(+). M. abscessus and M. chelonae ClpC1 plasmids were obtained from Genscript (Piscataway, NJ) using M. tuberculosis *clpC1* as a DNA template for mutagenesis. All recombinant plasmids were transformed into BL21-Gold (DE3) expression-competent E. coli (Agilent Technologies, Santa Clara, CA) for isopropyl-β-d-thiogalactopyranoside-induced protein expression at 37°C. Plasmid sequences were confirmed with Sanger DNA sequencing performed by the Research Resource Center at University of Illinois at Chicago (UIC). Frozen cells were lysed by a 30-min incubation on ice with lysozyme (1 mg/ml), benzonase nuclease (3 U/ml; Novagen), and a complete protease inhibitor tablet (EDTA free; Roche). After centrifugation, the C-terminal His-tagged protein was purified from the cell lysate with a nickel-nitrilotriacetic acid (Ni-NTA) spin column (Qiagen) preequilibrated with buffer A (20 mM phosphate [pH 8.0], 300 mM NaCl). The column was washed with buffer A plus 20 mM imidazole, and the protein was eluted in buffer A with 500 mM imidazole. Purity was evaluated by SDS-PAGE, and the protein was shown to migrate according to an approximate size of 95 kDa. The protein concentration was determined at *A*_280_ using the calculated extinction coefficient (ε_280_ = 35,870 M^−1^ cm^−1^), and then the buffer was exchanged (Zebra spin desalting column) into PBS with 15% glycerol and the protein was stored at −80°C.

### Analysis of cyclopeptide binding affinity to mycobacterial ClpC1 by SPR.

Each ClpC1 enzyme was immobilized on a CM5 sensor chip using standard amine coupling at 25°C with running buffer PBS-P (20 mM phosphate, 137 mM NaCl, 27 mM KCl, 0.05% surfactant P-20 [pH 7.4]) using a Biacore T200 instrument (GE Healthcare Bio-Sciences, Pittsburgh, PA). Unmodified blank surface was used on flow channel 1 as a control. ClpC1 enzymes were diluted with 10 mM sodium acetate (pH 4.0) to 100 μg/ml and immobilized to flow channels 2, 3, and 4 after sensor surface activation with 1-ethyl-3-(3-dimethylaminopropyl) carbodiimide hydrochloride (EDC)/N-hydroxy succinimide (NHS) followed by ethanolamine blocking on unoccupied surface area. Binding of RUFI, ECU, and CYMA to wild-type full-length ClpC1 from M. tuberculosis, M. abscessus, and M. chelonae were monitored in SPR binding buffer (PBS-P, 0.5 mM Tris(2-carboxyethyl)phosphine hydrochloride [TCEP], 2% DMSO) at 25°C and a flow rate of 30 μl/min. A series of increasing concentrations of the compounds were run over the surface of the immobilized full-length ClpC1 proteins. Data were double referenced with blank-channel and zero-concentration responses.

Kinetic association rate (*k*_a_) and dissociation rate (*k*_d_) constants were determined by fitting globally to the 1:1 Langmuir model embedded in the Biacore T200 evaluation software (v3.0). The *K_D_* (equilibrium dissociation constant) values were calculated from the determined rate constants (*K_D_* = *k*_d_/*k*_a_). Response units at each concentration were also measured during the equilibrium phase for steady-state affinity fittings, and the *K_D_* values were determined by fitting the data to a single rectangular hyperbolic curve equation (equation 1), where *y* is the response, *y*_max_ is the maximum response, and *x* is the compound concentration.

(1) $$y=\frac{y_{\text{max}}\times x}{K_{D}+x}$$

### Purification of recombinant ClpC1, ClpP1, and ClpP2 for functional assays.

Recombinant ClpC1 was expressed in E. coli Rosetta2 (DE3) (Novagen). Recombinant ClpP1 and ClpP2 were expressed in E. coli BL21(DE3) (Novagen). Purification of all proteins was carried out by Ni-*tris*-carboxymethyl ethylene diamine (TED) chromatography (Macherey-Nagel, Oensingen, Switzerland) following the user instruction manual. Briefly, E. coli cells were harvested by centrifugation (6,000 × *g*, 30 min, and 4°C) and broken for 10 min on ice using a sonicator (Branson, Emerson Industrial Automation, Danbury, CT). The soluble fraction was collected after centrifugation (12,000 × *g*, 30 min, and 4°C) and then passed through a 0.2-μm syringe filter for complete clearance of aggregates. After purification, the purity of the proteins was monitored by electrophoresis using SDS-PAGE. The protein concentration was determined using the bicinchoninic acid (BCA) protein assay reagent (Pierce Chemical Co., Dallas, TX) with standard bovine serum albumin.

### Measurement of ATPase activity of recombinant ClpC1.

The measurement of ClpC1 ATPase activity was carried out by the BIOMOL Green modified malachite green assay (51). The reaction mixture was prepared in assay buffer (100 mM Tris, 200 mM KCl, 8 mM MgCl_2_ [pH 7.5]), and the final volume of the reaction mixture was 50 μl. In the reaction mixture, the final concentrations of ClpC1 and ATP were 1 μM and 100 μM, respectively. For the measurement of the ATPase activity by treatment with ECU and RUFI, each compound, dissolved in DMSO, was added at 0.1, 1.0, and 10 μM as the final concentrations in each well of a transparent 96-well plate. To avoid any effect of protein denaturation by DMSO, 1 μl of compound solution was added to the reaction mixture. The reaction mixture was incubated at 37°C for 1 h, and then 50 μl of BIOMOL Green solution (Enzo Life Science, Farmingdale, NY) was added to the reaction mixture. After incubation for 10 min at room temperature, the OD of the reaction mixture was measured at 620 nm on an Infinite 200 PRO (Tecan, Switzerland). The concentration of free phosphate released from ATP by ClpC1 was calculated using a standard curve according to the concentration of free phosphate.

### Measurement of proteolytic activity.

Proteolytic activity of ClpC1, ClpP1, and ClpP2 was monitored by degradation of fluorescein isothiocyanate (FITC)-casein (Sigma-Aldrich) as the substrate (52). The reaction mixture was prepared in buffer used in the assay for ClpC1 ATPase activity, and the final volume of the reaction mixture was 100 μl. In the reaction mixture, the final concentrations of ClpC1, ClpP1, and ClpP2 were 1, 2, and 2 μM, respectively. In addition, 100 μM ATP and 1 μM FITC-casein were added to each reaction mixture. For the measurement of the degradation of FITC-casein by the ClpC1, ClpP1, and ClpP2 complexes in the presence of ECU and RUFI, each compound, dissolved in DMSO, was added at 0.1, 1.0, and 10 μM in each well of the black 96-well plates with a transparent bottom. To avoid protein denaturation by DMSO, only 2 μl of the compound solution was added to the reaction mixture. The reaction mixture was incubated at 37°C for 1 h, and then the fluorescence level of FITC-casein was measured at 485-nm excitation and 535-nm emission on an Infinite 200 PRO instrument (Tecan, Switzerland).

### Classification of *Streptomyces* sp. MJM3502 by 16S rRNA gene sequence.

*Streptomyces* sp. MJM3502 genomic DNA was extracted following the protocol of CoreBio (Republic of Korea) genomic DNA isolation kit. The 16S rRNA gene was amplified and sequenced by using the universal primers 27F (forward; 5′-AGAGTTTGATCATGGCTCAG-3′) and 1492R (reverse; 5′-AAGGAGGTGATCCARCCGCA-3′. The sequence was aligned with the closest *Streptomyces* strains in BLAST using CLUSTAL X (53), and the phylogenetic tree was constructed by the neighbor-joining method (54) using MEGA4.0 software (55). The topology of the phylogenetic tree was calculated by 1,000 bootstrap replications (56).

### Mouse pharmacokinetic study.

Healthy female BALB/c mice were administered a single dose (5 mg/kg) of RUFI intravenously (i.v.; tail vein). RUFI was formulated using 10% ethanol (EtOH), 40% 10% (2-hydroxypropyl)-β-cyclodextrin (HP-β-CD) solution, and 50% PEG 400. RUFI was first dissolved in 0.102 ml of EtOH, vortexed and sonicated until clear, and then diluted with 0.508 ml of PEG 400 with additional vortex treatment and sonication until clear. Finally, 0.406 ml of 10% HP-β-CD was added to the solution. Then 20 μl of this formulation was pipetted into a 10-ml volumetric flask, and methanol (MeOH) was added to a final concentration of 1 mg/ml. The accuracy of the formulation concentration was verified via high-performance liquid chromatography-tandem mass spectrometry (HPLC-MS/MS) (AB Sciex 6500+; SCIEX, Framingham, MA) by electrospray using multiple reaction monitoring (MRM; 1065.63/1047.70) in positive ion mode. LC was performed with a Kinetex EVO C_18_ column (50 mm by 3.00 mm [inside diameter], 2.6-μm particle size, 100-Å pore size) using a gradient of 0.1% formic acid (FA) in water–0.1% FA in acetonitrile (ACN) (25% ACN to 95% ACN in 1.5 min) at a flow rate of 0.8 ml/min.

Blood samples (10 μl) were collected via saphenous vein puncture at 5, 15, and 30 min and 1, 2, 4, 8, and 25 h. To each of the blood samples collected, 2.0 μl of MeOH was added followed by 200 μl of a solution of 5 ng/ml of terfenadine (internal standard) in MeOH. Samples were then vortexed for 1 min and centrifuged at 4,000 rpm for 15 min. The supernatant was collected and diluted with water, 2× (vol/vol) for HPLC-MS/MS analysis, as described above for the formulation determination. A standard curve was determined by using RUFI spiked into untreated mouse plasma.

## Supplementary Material

Supplemental file 1
