# Supplementary material for: Rufomycin Targets ClpC1 Proteolysis in Mycobacterium tuberculosis and M. abscessus
Source: Antimicrob Agents Chemother. 2019 Feb 26;63(3):e02204-18. doi: 10.1128/AAC.02204-18 (PMC6395927; doi:10.1128/AAC.02204-18)

Rufomycin Targets ClpC1 Proteolysis in Both *Mycobacterium tuberculosis* and *M. abscessus*

Mary P. Choules,[a,b] Nina M. Wolf,[a] Hyun Lee,[b,c] Jeffrey R. Anderson,[a§] Edyta M. Grzelak,[a] Yuehong Wang,[a] Rui Ma,[a] Wei Gao,[a,b*] James B. McAlpine,[a,b] Ying-Yu Jin,[d] Jinhua Cheng,[e] Hanki Lee,[d] Joo-Won Suh,[d,e] Nguyen Minh Duc,[d] Seungwha Paik,[f,g] Jin Ho Choe,[f,g] Eun-Kyeong Jo,[f,g] Chulhun L. Chang,[h] Jong Seok Lee,[i] Birgit U. Jaki,[a,b] Guido F. Pauli,[a,b] Scott G. Franzblau,[a] and Sanghyun Cho[a] #

Institute for Tuberculosis Research[a], Department of Medicinal Chemistry & Pharmacognosy[b], and Center for Biomolecular Sciences[c], College of Pharmacy, University of Illinois at Chicago, Chicago, IL, USA; Center for Nutraceutical and Pharmaceutical Materials[d] and Division of Bioscience and Bioinformatics, College of Natural Science[e], Myongji University, Cheoin-gu, Gyeonggi-do, Republic of Korea; Department of Microbiology[f] and Infection Control Convergence Research Center[g], Chungnam National University School of Medicine, Daejeon, Republic of Korea; Department of Laboratory Medicine[h], Pusan National University Yangsan Hospital, Yangsan, Republic of Korea; International Tuberculosis Research Center[i], Changwon, Republic of Korea.

Keywords: *M. tuberculosis*, *M. abscessus*, ClpC1, cyclic peptide

#Address correspondence to Sanghyun Cho, jkcno1@uic.edu

§Present address: Jeffrey R. Anderson, AbbVie, Mettawa, IL, USA

*Present address: Wei Gao, Nature's Sunshine Products, Inc., Lehi, UT, USA

Figure S1. Phylogenic tree of 16s rDNA classification of strain MJM3502.

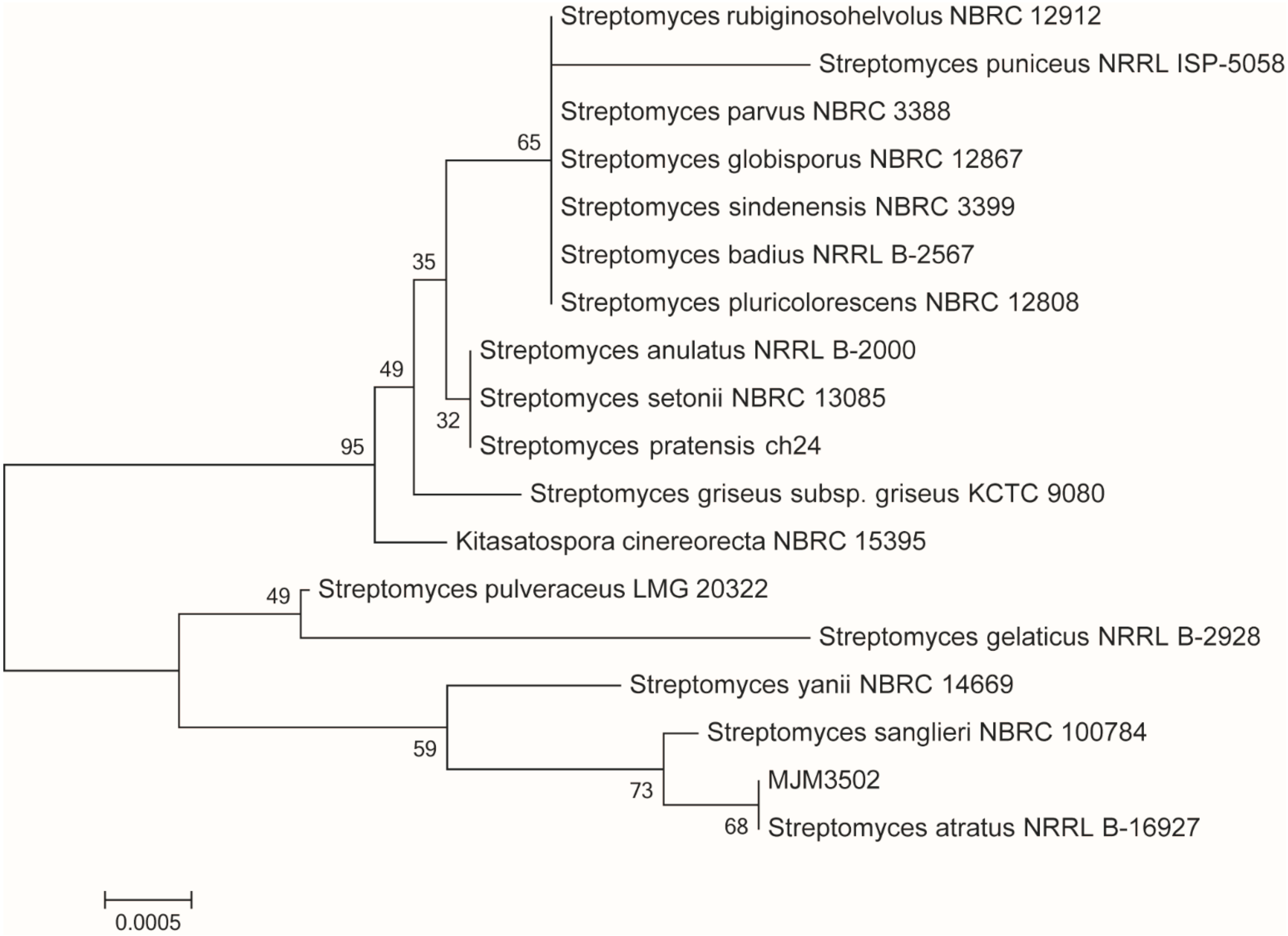

Supplement: Supplemental file 1 [file AAC.02204-18-s0001.pdf]
